# Short-Term PTH(1-34) Therapy in Children to Correct Severe Hypocalcemia and Hyperphosphatemia due to Hypoparathyroidism: Two Case Studies

**DOI:** 10.1155/2016/6838626

**Published:** 2016-11-13

**Authors:** Pooja E. Mishra, Betsy L. Schwartz, Kyriakie Sarafoglou, Kristen Hook, Youngki Kim, Anna Petryk

**Affiliations:** ^1^St John's Medical College, Bangalore, India; ^2^Pediatric Endocrinology, Park Nicollet, Minneapolis, MN, USA; ^3^Pediatric Endocrinology, University of Minnesota, Minneapolis, MN, USA; ^4^Pediatric Dermatology, University of Minnesota, Minneapolis, MN, USA; ^5^Pediatric Nephrology, University of Minnesota, Minneapolis, MN, USA

## Abstract

The standard treatment of hypoparathyroidism is to control hypocalcemia using calcitriol and calcium supplementation. However, in severe cases this approach is insufficient, and the risks of intravenous (i.v.) calcium administration and prolonged hospitalization must be considered. While the use of recombinant human parathyroid hormone 1-34 [rhPTH(1-34)] for long-term control of hypocalcemia has been established, the benefits of short-term rhPTH(1-34) treatment in children have not been explored. We report two patients with hypoparathyroidism treated with rhPTH(1-34). Patient 1 is a 10-year-old female with polyglandular autoimmune syndrome type 1. Patient 2 is a 12-year-old female with hypoparathyroidism after total thyroidectomy. Both patients showed poor response to i.v. and oral calcium and calcitriol, and patient 1 did not respond to phosphate binders. Patient 1 had rapid increase in serum calcium with a decrease in serum phosphate after a 3-day course of subcutaneous rhPTH(1-34). Patient 2 had normalization of calcium and phosphate levels after a 7-day course of rhPTH(1-34). These cases support a role for rhPTH(1-34) in the acute management of hypoparathyroidism in hospitalized patients to more rapidly correct hypocalcemia and hyperphosphatemia, shorten hospitalization, and reduce the need for frequent i.v. calcium boluses.

## 1. Introduction

Hypoparathyroidism may be due to a number of causes, including autoimmune destruction of parathyroid glands, accidental removal of or damage to the parathyroid glands following thyroid surgery, or genetic disorders such as 22q11.2 deletion. Hypoparathyroidism manifests as hypocalcemia and hyperphosphatemia with a low serum parathyroid hormone (PTH) level. Acute hypocalcemia is typically controlled with calcium and calcitriol supplementation. However, in some children these standard measures are insufficient, requiring prolonged hospitalization and even transfer to the pediatric intensive care unit (PICU) for intravenous (i.v.) calcium administration. Alternate therapeutic options for these children should be considered.

Use of recombinant human (rh) PTH(1-34) (teriparatide) has been documented in adults, with successful control of serum calcium and phosphate levels [1]. However, due to occurrence of osteosarcoma in rat toxicology studies of rhPTH(1-34) there is concern about its long-term use in a pediatric population [2].

Recent studies have demonstrated the advantage of using rhPTH(1-34) to control hypocalcemia in children for periods up to 3 years [3–5]. The two cases presented here suggest a role for rhPTH(1-34) in the acute management of hypocalcemia and hyperphosphatemia. This treatment can accelerate normalization of serum calcium and phosphate, reduce the need for i.v. calcium, and shorten the period of hospitalization.

## 2. Case Presentations

### 2.1. Patient 1

A 10-year-old female presented to dermatology clinic with a 4-year history of alopecia universalis and nail dystrophy, hypocalcemic seizures at 4 years of age (seizure-free on calcium and vitamin D supplements), and frequent thrush during infancy. A clinical diagnosis of autoimmune polyglandular syndrome type 1 was made and confirmed with a pathogenic mutation in the autoimmune regulator (*AIRE*) gene. She had a normal ACTH level and adequate peak cortisol level of 17.8 *μ*g/dL after 250 *μ*g of cosyntropin i.v.

**Figure 1:**
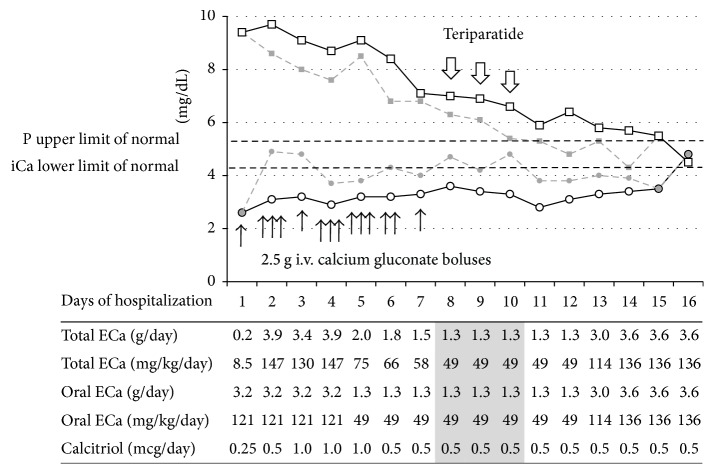
Trends in serum ionized calcium and phosphorus levels in a 10-year-old girl with hypoparathyroidism over the course of hospitalization. Minimum and maximum daily levels of iCa (circles and solid and dashed lines, resp.) and P (squares and dashed and solid lines, resp.) are shown. Black arrows indicate individual calcium gluconate boluses (2.5 g each). Open arrows point to once daily subcutaneous administration of teriparatide. Oral supplementation consisted of calcium carbonate except for Days 5–12 when calcium glubionate was given. ECa, elemental calcium.

A week later, she presented to the Emergency Department with tetany and a 3-day history of cramping in the hands and legs. Her serum iCa level was 2.3 mg/dL (reference range 4.4–5.2 mg/dL) and phosphate level was 9.2 mg/dL (reference range 2.9–5.4 mg/dL). She was given an i.v. bolus of 2.5 g calcium gluconate and 0.25 *μ*g of calcitriol before being transferred to PICU. Repeat testing showed persistent hypocalcemia and hyperphosphatemia and low PTH level (<3 pg/mL, reference range 12–72 pg/mL). She required multiple calcium gluconate boluses.  Figure 1 shows trends in iCa and P levels along with calcium and calcitriol doses. She received ergocalciferol 50,000 IU daily by mouth on days 2–4. On Day 5, her 25-hydroxyvitamin D level was normal (43 *μ*g/L, reference range 20–75 *μ*g/L) and ergocalciferol was discontinued.

On Day 3, due to persistently high serum phosphate, she was started on phosphate binder aluminum hydroxide (600 mg three times daily), and then Renagel (up to 9.6 g per day). Despite high dose calcium supplementation (up to 8 g of calcium carbonate per day) and renal protective measures, she continued to have hypocalcemia and hyperphosphatemia. On Day 8, she was started on rhPTH(1-34) (20 *μ*g once a day subcutaneously) and i.v. calcium boluses were discontinued. Within 48 hours, there was a significant rise in iCa and a drop in serum phosphate levels. rhPTH(1-34) was discontinued after 3 days of treatment. Hydrochlorothiazide (12.5 mg twice daily) was added on Day 13 to reduce renal calcium excretion because of nephrocalcinosis found on renal ultrasound. She was discharged on Day 15 with normal iCa (4.8 mg/dL) and P (4.5 mg/dL) levels a day later.

### 2.2. Patient 2

**Figure 2:**
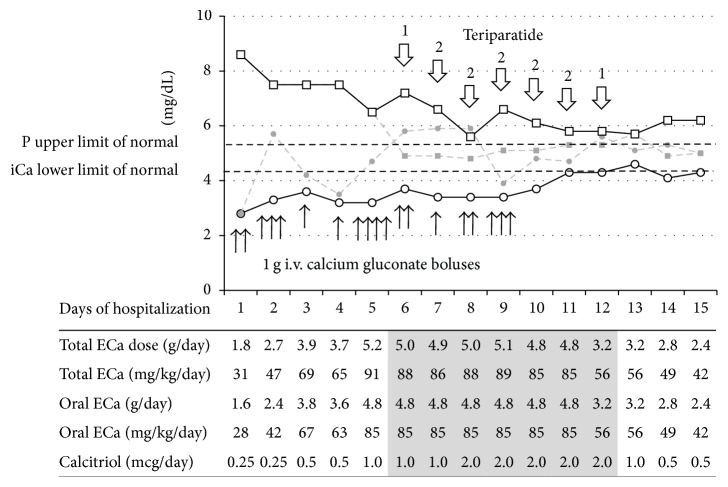
Trends in serum ionized calcium and phosphorus levels in a 12-year-old girl with hypoparathyroidism over the course of hospitalization. Minimum and maximum daily levels of iCa (circles and solid and dashed lines, resp.) and P (squares and dashed and solid lines, resp.) are shown. Black arrows indicate individual calcium gluconate boluses (1 g each). Open arrows point to days of subcutaneous administration of teriparatide with the numbers above the arrows indicating once daily (1) or twice daily (2) injections. Oral supplementation consisted of calcium carbonate except for Days 2-3 when calcium citrate was also given. ECa, elemental calcium.

Patient 2 is a 12-year-old girl diagnosed with Graves' disease five weeks prior to admission, treated with methimazole. Two weeks later she was found to have a thyroid nodule and was diagnosed with papillary thyroid cancer. She underwent total thyroidectomy with central neck dissection without removal of the parathyroid glands. Following surgery she was started on 112 mcg of L-thyroxine daily, calcium carbonate 1000 mg four times a day, and calcitriol 0.25 *μ*g daily. She presented to the Emergency Department five days later with muscle spasms, irritability, and difficulty in swallowing. Laboratory evaluation revealed severe hypocalcemia (iCa 2.8 mg/dL, Figure 2) and hyperphosphatemia (8.6 mg/dL). PTH level was <0.3 pg/mL. Free T4 level was normal (0.99 ng/dL, reference range 0.76–1.46 ng/dL), but TSH was still suppressed at <0.01 mU/L (reference range 0.4–4.0 mU/L).

She was given two i.v. boluses of 1 g calcium gluconate and was transferred to PICU for further management. She required repeated i.v. calcium boluses in addition to oral supplementation with increasing doses of calcium carbonate (up to 12 g per day). The dose of calcitriol was gradually increased to 2 *μ*g per day. Her 25-hydroxy-vitamin D level was 27 *μ*g/L. She received 50,000 IU of ergocalciferol on Days 4 and 11, cholecalciferol 1,000 IU daily on Days 3–7, and then 4,000 IU daily starting on Day 8. Due to persistent signs and symptoms of hypocalcemia (prominent Chvostek sign, paresthesias, and QTc prolongation), rhPTH(1-34) treatment was started on Day 6 at a dose of 20 *μ*g once a day subcutaneously. The same day, serum phosphate level decreased rapidly from 7.2 to 4.9 mg/dL, and serum iCa increased from 3.7 to 5.8 mg/dL. The dose of rhPTH(1-34) was temporarily increased to 20 *μ*g twice daily on Days 7–11 because the effect diminished before the next scheduled dose. After 4 days of rhPTH(1-34) treatment, the patient no longer required i.v. calcium boluses. With near normalization of serum calcium and phosphate levels, rhPTH(1-34) was discontinued on Day 12. On discharge (Day 15), her iCa level was 4.8 mg/dL and phosphate level was 5.6 mg/dL.

## 3. Discussion

Standard therapeutic approaches in patients with hypoparathyroidism include calcium and vitamin D supplementation. Persistent severe hypocalcemia requires i.v. calcium gluconate administration in the form of i.v. boluses or as a continuous infusion. The latter may have to be continued for a week to ensure enterocyte recovery and adequate intestinal absorption of oral calcium and carries significant risks (cardiac arrhythmia, extravasation, and thrombophlebitis). Moreover, traditional therapy is not causative because it does not replace the deficient hormone.

While there is accumulating evidence for the benefits of rhPTH(1-34) replacement in children on a long-term basis [3], these two cases illustrate that a short-term treatment may also benefit children with refractory hypoparathyroidism by allowing faster normalization of calcium and phosphorus levels and shortening hospitalization, particularly when continuous i.v. calcium infusion is neither feasible nor effective. Similar benefits of short-term rhPTH(1-34) therapy have been demonstrated in adults with postsurgical hypoparathyroidism [6]. Certain patient populations are at a particularly high risk for severe hypocalcemia, for example, after thyroidectomy for thyroid cancer or Graves' disease, partly due to “hungry bone syndrome” in the latter [7], as illustrated by Patient 2.

Twice daily dosing of rhPTH was required in Patient 2 due to significant excursions in serum calcium levels, consistent with previous data showing less variation on a twice daily regimen compared to a once daily injections [8]. It remains to be determined if a longer acting rhPTH(1-84) molecule with favorable long-term safety and efficacy in adults [9] would benefit children as well.
